# Characteristics of patients seeking fertility care in a low-income setting

**DOI:** 10.5935/1518-0557.20230073

**Published:** 2024

**Authors:** Miriam Tarrash, Olutunmike Kuyoro, Randi H. Goldman, Christine Mullin

**Affiliations:** 1 Northwell Health Fertility, North Shore University Hospital/Donald and Barbara Zucker School of Medicine at Hofstra/Northwell, Manhasset, NY, USA

**Keywords:** access to care, health disparities, medicaid, fertility workup

## Abstract

**Objective:**

Patients face challenges accessing fertility treatment due to barriers such as financial burdens, delayed referral to Reproductive Endocrinologists (REI), low medical literacy, language barriers and numerous other health disparities. Medicaid in New York offers coverage for office visits, blood tests, hysterosalpingograms (HSGs), and pelvic ultrasounds for infertility. The aim of this study is to delineate the characteristics of this underserved population and determine their ability to complete the initial fertility workup.

**Methods:**

This was a retrospective study of all patients seeking fertility care at a single resident/fellow REI clinic in New York from September 2020 - January 2022.

**Results:**

During the study period, 87 patients (avg age = 35.2y) sought care at the resident/fellow clinic over 126 appointments. The majority of patients had Medicaid insurance and most primary languages spoken included English (70.1%), Spanish (21.8%), and Bengali (3.4%). Documented Race was comprised of mostly Other (46%), African American (21.8%), Asian (17.2%), and White (11.5%). The majority of patients completed a lab workup (70-80%). Fewer patients underwent a scheduled HSG (59.8%) and patients’ partners completed a semen analysis (SA) (27.6%). Overall, there was a significant difference in the ability to complete the initial infertility workup (lab tests vs. HSG vs. SA) across all groups regardless of age, insurance type, primary language spoken, race and ethnicity (*p*<0.05).

**Conclusions:**

Completing the fertility workup, particularly the male partner workup and imaging studies, can present challenges for underserved patients with infertility. Understanding which patient characteristics and societal factors restrict access to fertility care requires further investigation to improve access to fertility care in underserved communities.

## INTRODUCTION

Patients face challenges accessing fertility treatment due to barriers such as financial burdens, delayed referral to reproductive endocrinologists (REI), low medical literacy, language barriers, structural racism, difficulty attending appointments, and numerous other health disparities (Goodman *et al*., 2012; Ho *et al*., 2017; Farland *et al*., 2016; Paulson *et al*., 2016; Brown *et al*., 2019; Churchwell *et al*., 2020). Women seeking fertility care from low resource, predominantly immigrant communities have greater disparities in fertility knowledge and overall lower health literacy compared to women from high resource clinical settings (Hoffman *et al*., 2020; Letourneau *et al*., 2012; Stormacq *et al*., 2019). Studies have demonstrated that education level and household income are associated with increased total amount of money spent on fertility care, which impacts the likelihood of live birth (Smith *et al*., 2011). Poorer reproductive outcomes and racial/ethnic disparities in IVF outcomes have been found in all minority groups when compared with Caucasian women (Fujimoto *et al*., 2010; Wellons *et al*., 2012; Feinberg *et al*., 2007; Quinn & Fujimoto, 2016). In the US, minority ethnic groups and Black women have lower assisted reproductive technology (ART) utilization rates, even after accounting for socioeconomic and country-specific factors (Quinn & Fujimoto, 2016; Dieke *et al*., 2017; Shapiro *et al.,* 2017; Armstrong & Plowden, 2012). Understanding these disparities is crucial in order to reduce this discrepancy and improve access to fertility care for underserved communities.

**Figure 1:**
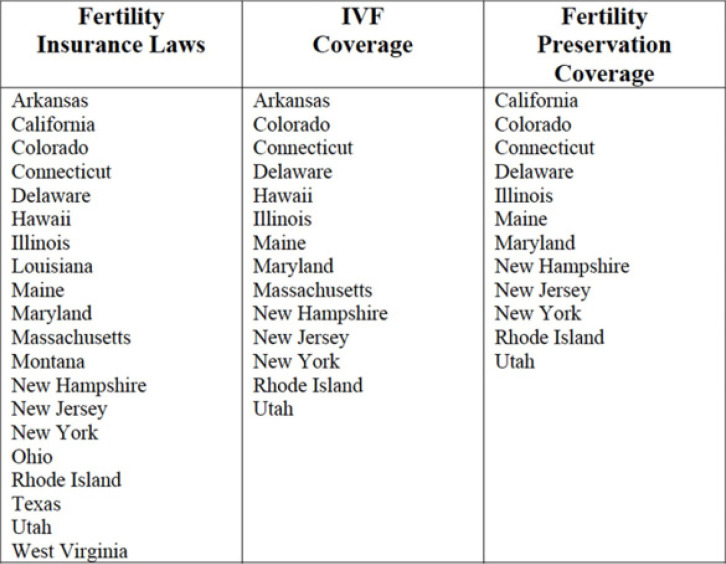
Differences in mandated states fertility insurance coverage by state.

Access to fertility treatment in the United States is not uniform because of state level variations in health insurance coverage. Some states have mandated insurance coverage for fertility treatments including IVF, while others do not provide such coverage. As of June 2022, 20 states have passed fertility insurance coverage laws mandating that private insurers provide coverage for some fertility treatments, although not all mandates require coverage for ART. Fourteen of those states’ laws include IVF coverage and 12 states have fertility preservation laws for iatrogenic (medically induced) infertility (Figure 1) according to the Insurance coverage by state article by Resolve (2022).

Insurance mandates for infertility treatments have been associated with greater use of ART and state-level differences in ART utilization, likely explained by differences in health insurance coverage (Dieke *et al*., 2017). States with mandated health insurance coverage for fertility treatment have three times higher use of IVF and a lower incidence of multiple births when compared to states without mandated coverage (Jain *et al*., 2002; Henne & Bundorf, 2008; Hamilton & McManus, 2012; Boulet *et al*., 2015). Expanding insurance coverage may benefit vulnerable populations such as those undergoing fertility preservation for cancer, couples who carry a genetic disorder, LGBTQIA+, and uninsured patients.

Globally, ART utilization varies widely and has been used as a marker for access to ART (Lass & Lass, 2021; Dyer *et al*., 2020). Access to ART, in part, is restricted due to strict regulations in some countries. For example, in several countries including Algeria, Bahrain, Costa Rica, Egypt, Hong Kong, Jordan, Lebanon, Lithuania, Libya, Maldives, Oman, Pakistan, Philippines, Qatar, Saudi Arabia, Syria, Tajikistan, Tunisia, Turkey, United Arab Emirates, and Yemen, laws prohibit sperm donation. Moreover, egg donation is not available in countries such as China, Germany, Japan, Turkey and Saudi Arabia according to the International Federation of Fertility Societies (2016). Other countries have restrictions regarding the ability to use preimplantation genetic diagnosis (PGD), cryopreservation and other ART services (Bayefsky, 2016). Worldwide, such strict regulations add barriers to access ART care. Lastly, Asia and Africa have the lowest rates of ART utilization globally (Dyer *et al*., 2020). Even though Africa has higher rates of STIs, postpartum and post abortion infections that lead to increased rates of infertility, many patients needing fertility services are not able to access care. Cultural acceptance of ART, influenced by education, is a primary catalyst of ART treatment use in Europe, beyond the expected rate based on the country’s wealth, demographics and religious makeup (Präg & Mills, 2017). These country-specific differences lead to fertility tourism requiring patients to cross borders to meet their needs.

According to the American Society for Reproductive Medicine (ASRM) the infertility evaluation should include an assessment of ovulatory status, patency of the female reproductive tract via hysterosalpingogram (HSG) or saline infusion sonohysterogram, and semen analysis (SA) of the male partner (Practice Committee of the American Society for Reproductive Medicine, 2021). Medicaid in New York offers coverage for office visits, blood tests, HSGs, pelvic ultrasounds and partner SA for infertility. Within our hospital system, the uninsured and underinsured patients are referred to a resident and fellow REI clinic with limited available treatment options. The aim of this study is to delineate the characteristics of this underserved patient population and assess their ability to complete the initial fertility workup.

## MATERIAL AND METHODS

### Study design

This was a retrospective study of all patients seeking fertility care at a single resident and fellow run REI clinic in New York from September 2020 - January 2022. The data was collected from the electronic medical record and the primary language spoken, race, and ethnicity were obtained from the self-reported patient profile. The retrospective review and analysis of data collected was approved by the Northwell Institutional Review Board.

### Patients

A total of 87 different patients with 126 appointments were included. Patients were stratified based on age, insurance, primary language spoken, race, and ethnicity. There were no patients excluded from this study.

### Outcomes

The primary outcomes were comparisons of completion of blood work, hysterosalpingogram (HSG), and semen analysis (SA). Patient specific variables were collected including patient age, insurance, primary language spoken, race, and ethnicity.

### Statistical analysis

Chi-square analyses were used to compare differences in patients’ ability to complete the initial infertility workup. *p*<0.05 defined statistical significance.

## RESULTS

During the study period, 87 patients sought care at the resident and fellow run clinic over 126 scheduled appointments. 66.7% of patients attended their scheduled appointment. The average age at presentation or first appointment scheduled with our fertility clinic was 35.2±5.9 with the patient age ranging from 23 to 46 years old in this study.

80.5% of patients had Medicaid, 12.2% used Sliding Scale Health Center, and 2.3% had no health insurance. Most primary languages spoken included English (70.1%), Spanish (21.8%), and Bengali (3.4%). Other languages consisted of American Sign Language (1.1%), Creole (1.1%), Korean (1.1%) and Punjabi (1.1%). Documented Race was comprised of Other (46%), African American (21.8%), Asian (17.2%), White (11.5%), Native Hawaiian (1.1%), and Unknown (2.3%). Approximately 35% of the population identified as Hispanic or Latino.

The majority of patients completed a lab workup (70-80% obtained AMH, which was typically drawn at the time of the initial visit, and day 3 labs). Fewer patients underwent a scheduled hysterosalpingogram (59.8%, of which 40.4% had abnormal findings). A smaller proportion of patients’ partners completed a SA (27.6%, of which 50% had abnormal findings).

Overall, there was a significant difference in the ability to complete the initial infertility workup (lab tests *vs*. HSG *vs*. SA) across all groups regardless of age, insurance type, primary language spoken, race and ethnicity (*p*<0.05). Each group had more patients complete bloodwork when compared to HSG and SA (Table 1).

**Table 1:** Completion of infertility workup by patient characteristics.

Characteristics		% Complete			p value
		Blood	HSG	SA	
Age	<35	84.2	63.2	26.3	**<0.0001**
	≥35	77.6	59.2	28.6	**<0.0001**
Insurance	Medicaid	77.1	58.6	28.6	**<0.0001**
	Sliding scale	93.3	60	26.7	**<0.0001**
Language	English	78.7	60.7	26.2	**<0.0001**
	Spanish	84.2	63.2	21.1	**<0.0001**
	Other	85.7	42.9	57.1	**0.0005**
Race	African American	78.9	42.1	10.5	**<0.0001**
	Asian	86.7	66.7	40	**0.0002**
	White	70	40	40	**0.002**
	Other	81.4	69.8	27.9	**<0.0001**
Ethnicity	Hispanic	90	70	26.7	**<0.0001**
	Non-Hispanic	78.2	56.4	29.1	**<0.0001**
**Total**		80.5	60.9	27.6	**<0.0001**

**Figure 2:**
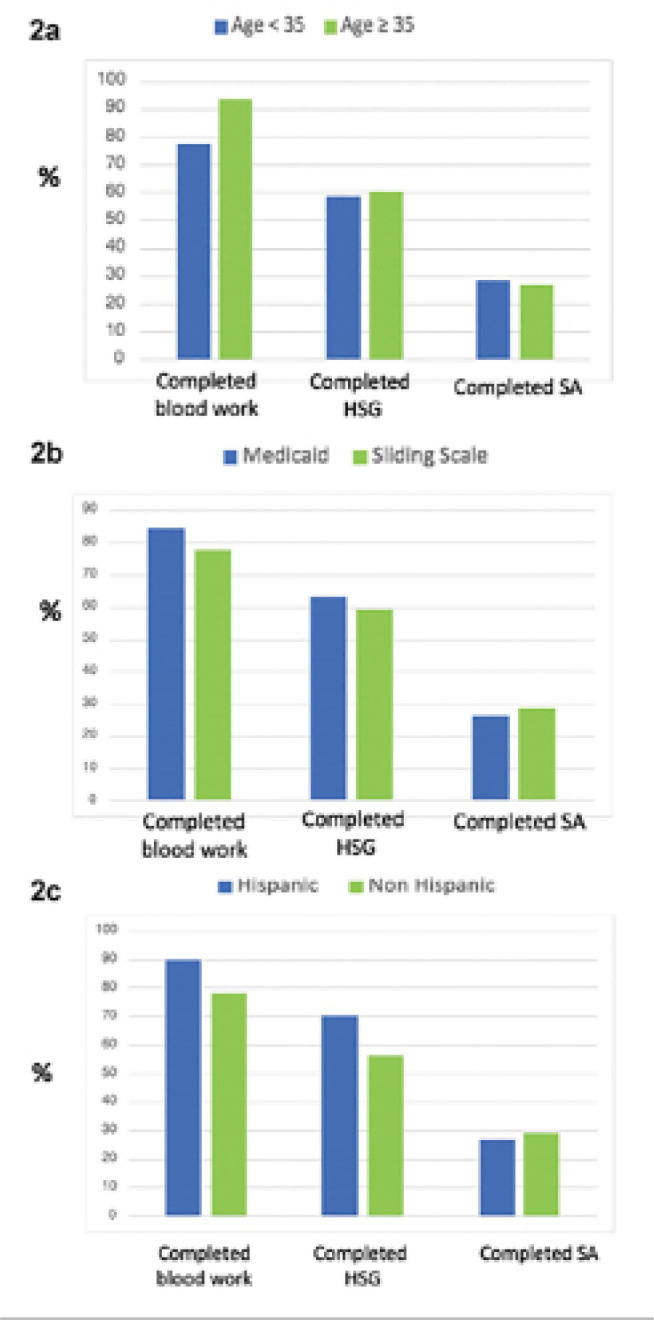
Completion of infertility workup by age (2a), insurance (2b), and ethnicity (2c).

The ability to complete each of the 3 tests in the initial infertility workup (bloodwork, HSG, SA) did not differ according to by age <35y *vs*. ≥35y), insurance (Medicaid *vs*. Sliding Scale), or ethnicity (Hispanic *vs*. Non-Hispanic) (Figures 2a, 2b, and 2c).

**Figure 3:**
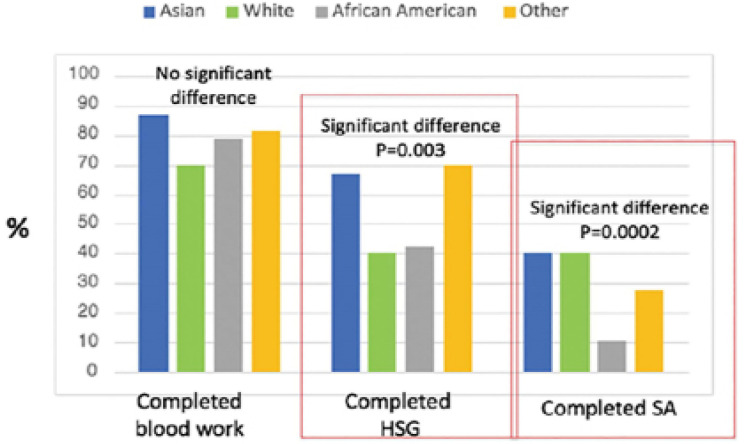
Completion of infertility workup as compared by race.

Completion of HSG and SA was significantly different when stratified by race. More Asians and Other races completed HSG as compared to White and African American races (*p*=0.003) whereas fewer partners of African American patients completed SA when compared with Asian, White and Other races (*p*=0.0002) (Figure 3).

**Figure 4:**
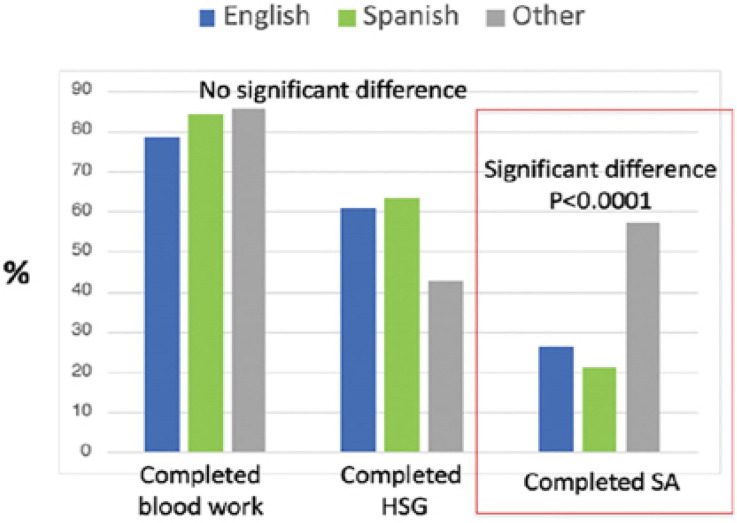
Completion of infertility workup by as compared by language.

More partners of patients speaking Other languages completed the SA as compared with English and Spanish speakers (*p*<0.0001) (Figure 4), though total sample size in this group was small. Other languages include: 1 ASL, 3 Bengali, 1 Creole, 1 Korean, 1 Punjabi.

## DISCUSSION

Completing the fertility workup, particularly the SA and HSG, can present challenges for underserved patients with infertility. Those patients with an infertility diagnosis may have numerous barriers to fertility access to care such as low socioeconomic status, marital status, employment, transportation, and type of health insurance (Farland *et al*., 2016; Paulson *et al*., 2016). These obstacles are magnified in immigrant communities (Hoffman *et al*., 2020; Hasstedt *et al.*, 2018). Low literacy has been associated with poor health outcomes and reduced use of health resources for patients (Boulet *et al*., 2015).

Understanding patients’ personal factors hindering arrival to appointments such as access to affordable transportation, childcare, employment restraints, and other health disparities may help to coordinate solutions. For example, our resident and fellow run fertility clinic is only available one afternoon every 3 weeks with limited available appointment times. As a result, patients often book appointments that are months away based on availability. Increasing appointment availability will create a greater access to care for all patients.

Our results emphasized particularly low rates of SA completion among this population. Men may have low self-esteem, guilt, frustration and embarrassment with the diagnosis of male factor infertility and therefore take longer to present for care (Warchol-Biedermann, 2021; Balasubramanian *et al*., 2018). In our clinic, this lapse in care appears to be more of an access issue and psychosocial factor rather than a financial one, as diagnostic testing is covered for nearly all patients. In the future, perhaps FDA approved home diagnostic testing and increased opportunities for telehealth visits could help improve access to care and completion of SA (Grens *et al*., 2022).

According to our study, Asians and other races completed HSG at higher rates, while partners of African American patients completed SA at lower rates. More partners of patients speaking other languages completed the SA. Literature has highlighted the difficulties that African American patients face while pursuing high quality fertility care with numerous health disparities (Butts, 2021; Seifer *et al*., 2010). Furthermore, studies of ART cycles have found lower clinical pregnancy rates in Asian women and lower live birth rates in African American, Hispanic and Asian women with no clear explanations (Shapiro *et al*., 2017; Armstrong & Plowden, 2012; Purcell *et al*., 2007). However, no study to date has specifically evaluated race or language as they relate to the ability to complete the fertility workup. Interestingly, one study characterized factors impacting exposures to male infertility (Noncent *et al*., 2017). African American men self-reported learning about infertility primarily from television or physicians. Jewish men were most likely to know someone who has undergone infertility treatment and Asian men were least likely to know family members that have undergone infertility treatment (Noncent *et al*., 2017). Limitations of studies evaluating fertility treatment outcomes include missing data categorizing patients’ race and ethnicities. Our understanding of how race and ethnicity influences ART outcomes could be dramatically improved if accurate reporting increased (Wellons *et al*., 2012). Further research on this topic is necessary to better interpret these findings.

Limitations to our study include a relatively small sample size. Our data reflect the experiences of low-income patients seeking fertility care at one clinic in New York but may not be generalizable to populations in other geographic areas with differing insurance mandates and laws regarding fertility care. Race and ethnicity were self-reported measures in our study, which could lead to error in these results. In addition, our data may not represent those patients who haven’t been referred or sought out care with REI specialists.

All patients with infertility or requiring fertility preservation deserve to understand their reproductive options and make informed decisions. Insurance coverage trends should continue to lean in the direction of patients and their needs to ensure equal access to care and improved fertility outcomes (Seifer *et al*., 2018). Laws that mandate health insurance coverage for fertility services may decrease disparities in the outcomes of infertile couples (Jain *et al*., 2002; Henne & Bundorf, 2008).

## CONCLUSION

Completing the fertility workup, particularly imaging studies and semen analysis, presents the biggest challenges for this underserved patient population, despite the majority of patients having insurance coverage for such diagnostic tests. Hence, access to care may be less of a financial limitation than a societal barrier. Insurance coverage trends should continue to lean in the direction of patients to ensure equal access to fertility care. As such, understanding which patient characteristics and societal factors restrict access to fertility care requires further investigation, in order to improve access to fertility care in such underserved communities.
